# Case Report: Polyarteritis nodosa or complicated Henoch-Schonlein purpura (IgAV), a rare case

**DOI:** 10.12688/f1000research.13295.2

**Published:** 2018-04-16

**Authors:** Sajad Hasanzadeh, Seyedeh Maryam Alavi, Elahe Masnavi, Saeid Jokar, Maryam Rohani

**Affiliations:** 1 Department of Internal Medicine, Yasouj University of Medical Sciences, Yasouj, Iran; 2 Department of Obstetrics and Gynecology, Yasouj University of Medical Sciences, Yasouj, Iran

**Keywords:** Polyarteritis nodosa, Henoch-Schonlein purpura (Immunoglobulin A-associated vasculitis), vasculitis

## Abstract

**Background**: Polyarteritis nodosa (PAN) is a vasculitis that affects medium-sized arteries. PAN is a rare disease and requires a high clinical suspicion for diagnosis. PAN and HSP (newly named Immunoglobulin A-associated vasculitis) have narrowing differential diagnosis. Here, we reported a case of PAN.

**Case presentation**: Our patient was a 65-year-old woman that came to hospital due to abdominal pain and skin lesion on the right upper and right lower extremities. All rheumatologic tests were negative. A biopsy of the skin lesion was reported as mild hyperkeratosis, slight spongiosis with intact basal layer. The dermis showed moderate to severe perivascular PMN infiltration with vessel wall degeneration and extravasation of RBCs. A colonoscopy reported diffuse mucosal erythema and erosions were seen in the rectum until 6cm of anal verge. An electromyogram test and nerve conduction velocity study of the upper extremities reported bilateral mild carpal tunnel syndrome, and in the right lower extremities mononeuritis multiplex could not be ruled out. Abdominopelvic CT scan reported diffuse wall thickening of terminal ileum associated with mesenteric fat and narrow enhancement of inferior Mesenteric artery with patchy filling defect. After evaluation, the patient received corticosteroid pulses plus cyclophosphamide.

**Conclusion**: Diagnosis and treatment of PAN are important and PAN should be considered in a patient with skin lesions and neurological impairment.

## Introduction

Polyarteritis nodosa (PAN) is a systemic vasculitis that mostly involves medium sized arteries, and sometimes involves small arteries ^1^. The prevalence of PAN is estimated to be 2 to 33 million individuals ^2, 3^. The annual incidence in some areas of Europe estimate 4.4 to 9.7 per million population ^4^. The diagnosis is most commonly made in middle-aged or older adults and increases with age, and its peak is in the sixth decade of life ^2^. Polyarteritis nodosa can mimic the clinical manifestations of Henoch-Schonlein purpura (HSP) that is newly named Immunoglobulin A-associated vasculitis (IgAV). It is difficult to differentiate between PAN and HSP (IgAV), at an early stage. If PAN is not diagnosed and treated at an early stage, it has a high morbidity ^5, 6^. Considering that PAN is a rare disease and requires a high clinical suspicion for diagnosis, here, we report a case of PAN and the reasoning behind its diagnosis in our patient.

## Case report

### Patient information

The patient was a 65 year old woman from Yasouj (south of Iran) that came to our hospital due to abdominal pain and skin lesion on right upper and right lower extremities, which were was mostly on the distal of extremities, for since 2 weeks preadmission. Other complaints of the patient were diarrhea, vomiting, chills, fever, and anorexia. The patient did not complain of arthralgia. In the past medical history, the patient had Diabetes Mellitus, hypertension, and Bell's palsy one week pre-admission (treatment with 40mg prednisolone QD).

### Clinical findings

**Figure 1.**
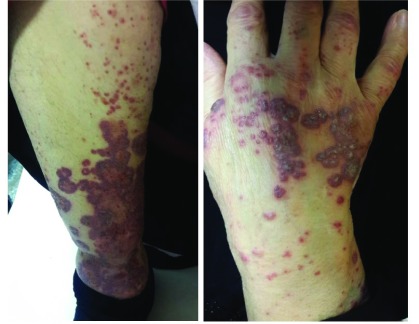
Palpable plaque in the erythematous and purpuric context.

On examination of the skin, the patient had palpable plaque in the erythematous and purpuric context with vesicular and bulla lesion on right upper and right lower extremities that mostly extended to the distal part ( Figure 1). An abdominal examination revealed mild tenderness in the epigaster. The Right lower extremities were warm and end pulses were normal. In active and passive motion of the joints had not painful movements. Neurologic exam of the right lower extremity revealed decreased sense and motor function (muscle power 4/5).

### Diagnostic assessment

Laboratory tests: HCV, HBV, HIV, ANA (antinuclear antibodies), cryoglobulin, anti-double-stranded DNA (dsDNA) antibodies, complement (C3 and C4), perinuclear antineutrophil cytoplasmic antibodies (P-ANCA and C-ANCA), all were normal. Urine analysis, Kidney performance (BUN and Creatinine) tests was normal, amylase and lipase levels were normal. ESR was 40mm/h (Normal under 20mm/h), occult blood one pluses positive, and hemoglobin was 11/9 g/L (Normal 13–16g/l).

*Skin biopsy:* Mild hyperkeratosis, slight spongiosis with intact basal layer. The dermis showed moderate to severe perivascular PMN infiltration with vessel wall degeneration and extravasation of RBCs. A diagnosis of a vasculitis leukocytoclastic variant (immunofluorescence is not available at our center).

Evaluation of patient anemia and GI tract were done via endoscopy and colonoscopy.

*Endoscopy:* Patchy erythematous lesions were observed.

**Figure 2.**
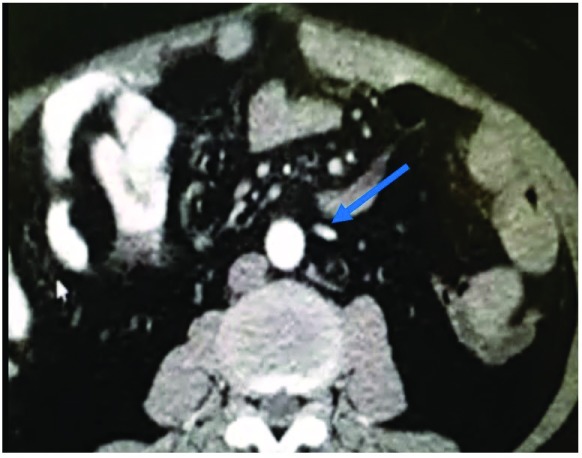
Abdominopelvic CT scan with IV contrast. Narrow enhancement of the inferior mesenteric artery can be observed (blue arrow).

*Abdominopelvic CT scan ( Figure 2)*: A 130mm of segment of terminal ileum had diffuse wall thickening (3–8mm) associated with mesenteric fat. Narrow enhancement of inferior mesenteric artery with patchy filling defect, poor enhancement of terminal branches. Therefore, suspicions were: 1)vasculitis, 2)mesenteric ischemia.

*Colonoscopy:* Diffuse mucosal erythema and erosions were seen in the rectum until 6cm of anal verge. Hemorrhoid without active bleeding in anus, few erythema and ophtus ulcer in cecum. Terminal ileum was not intubated. A diagnosis of a rectal erosion maybe due to vasculitis.

*Electromyogram test and nerve conduction velocity:* Upper extremities reported bilateral mild carpal tunnel syndrome, and in right lower extremities mononeuritis multiplex could not be ruled out.

*Echocardiography*: No evidence of any other disorder.

**Final diagnosis**: Vasculitis PAN or complicated HSP (IgAV)

Therapeutic intervention

The patient received 1000 mg methylprednisolone IV pulse daily for 3 days, and 750mg cyclophosphamide IV pulse every two weeks for 3 weeks.

### Follow-up and outcomes

After 24 hours of receiving corticosteroid pulses and cyclophosphamide, the symptoms of the patient subsided, with skin lesions going into remission. Currently, the patient is being treated with 50mg prednisolone daily, after 2 weeks, if there is no recurrence of patient symptoms we will taper off corticosteroids amount by 10%. We will reduce the dose of corticosteroids until we have control of patient symptoms, then we will make decisions depending on the patient’s condition.

## Discussion

Unlike other vasculitis’s such as microscopic polyarthritis or Wegener’s, PAN is not associated with ANCA ^7^. The organs most often affected in PAN are the skin, renal and GI tract. Cardiac involvement can manifest itself with hypertension, or even ischemic heart disease ^8^. In the skin, PAN may manifest by erythematous nodules, livedo reticularis, ulcer, bullous or vesicular eruption and purpura ^7, 9, 10^. Gastrointestinal symptoms that may be seen include abdominal pain, nausea, vomiting, melena, bloody or non-bloody diarrhea, and life-threatening gastrointestinal bleeding ^11^. One of the most common manifestations of patients with PAN is mononeuropathy multiplex that typically involves both motor and sensory deficits in up 70% of patients ^7, 12^. Some of the patients have sensorineural hearing loss ^13^ Most cases of PAN are idiopathic, although hepatitis B virus infection, hepatitis C virus infection, and hairy cell leukemia are important in the pathogenesis of some cases ^3, 4, 14, 15^. PAN can mimic the clinical manifestations of HSP (IgAV). It is difficult to differentiate between PAN and HSP (IgAV) at an early stage ^5^. The biopsy pattern helps to differentiate between PAN and HSP (IgAV); in tissue studies of HSP (IgAV) leukocytoclastic vasculitis in postcapillary venules together with IgA deposition is observed ^16^. As already mentioned, PAN is most commonly seen in middle-aged or older adults ^3^, while HSP (IgAV) is a childhood disease that occurs between the ages of 3 and 15 years ^17^. Neurologic manifestation in HSP (IgAV) is rare. Single reports and case series document neurologic manifestations including headaches, intracerebral hemorrhage, focal neurologic deficits, ataxia, seizures, and central and peripheral neuropathy in children with HSP (IgAV) ^18^. According to EULAR/PRINTO/PRES classification criteria, there was no renal failure, arthralgia and arthritis in this patient, but basis on other item, HSP (IgAV) could be diagnosis ^19^. In the present case, using clinical manifestations and laboratory tests, we excluded another differential diagnosis apart from PAN. Considering that PAN and HSP (IgAV) have narrowing clinical manifestation, we differentiated between the two diseases by age and neuropathy. However, although the diagnosis of the present patient is PAN, for a better diagnosis, immunofluorescence of the biopsy was needed, which is not available in our center. Finally, diagnosis and treatment of PAN are important. PAN should be considered in a patient with skin lesions and neurological impairment.

## Data availability

The data referenced by this article are under copyright with the following copyright statement: Copyright: © 2018 Hasanzadeh S et al.

Data associated with the article are available under the terms of the Creative Commons Zero "No rights reserved" data waiver (CC0 1.0 Public domain dedication). 

All data underlying the results are available as part of the article and no additional source data are required.

## Consent

Written informed consent was obtained from the patient for the publication of the patient’s clinical details and accompanying images.
