# Supplementary material for: Case Report: Polyarteritis nodosa or complicated Henoch-Schonlein purpura (IgAV), a rare case
Source: F1000Res. 2018 Apr 16;7:49. Originally published 2018 Jan 12. [Version 2] doi: 10.12688/f1000research.13295.2 (PMC5930546; doi:10.12688/f1000research.13295.2)
Supplement: Peer review history

# Peer review: Referee response for version 2

**DOI:** 10.5256/f1000research.15802.r33221

**Published:** 2018-05-01

**Authors:** Patricia Woo

**Affiliations:** 1 Division of Infection and Immunity, University College London, London, UK

The message is much clearer. Thank you. There are some typos, but I am happy to approve its indexing.

I have read this submission. I believe that I have an appropriate level of expertise to confirm that it is of an acceptable scientific standard.

# Peer review: Referee response for version 2

**DOI:** 10.5256/f1000research.15802.r33220

**Published:** 2018-04-17

**Authors:** Marco de Vincentiis

**Affiliations:** 1 Department of Oral and Maxillofacial Sciences, Sapienza University of Rome, Rome, Italy

The authors addressed my comments and questions. The article can be indexed in the present form.

I have read this submission. I believe that I have an appropriate level of expertise to confirm that it is of an acceptable scientific standard.

# Peer review: Referee response for version 1

**DOI:** 10.5256/f1000research.14428.r31810

**Published:** 2018-03-13

**Authors:** Patricia Woo

**Affiliations:** 1 Division of Infection and Immunity, University College London, London, UK

This is indeed a severe case of systemic vasculitis. The criteria used to classify the combined clinical and histological findings are not clearly referenced for discussion. The EULAR/PRINTO/PRES criteria published in 2016 are more discriminatory with the addition of IgA immunofluorescence for IgA associated vasculitis (formerly known as HSP). Unfortunately the authors were not able to perform this test and the skin histology did not describe a necrotizing vasculitis. The authors are correct that mononeuritis multiplex would be a separator, but the evidence in this case is more circumstantial as reported. The time line of Bells palsy is suggestive. The nerve conduction report is equivocal. Renal dysfunction or joint involvement were not mentioned. 

 Both conditions can respond well to prednisolone alone, or in combination with cyclophosphamide depending on the severity. 

 Since the case report is to highlight the overlapping features of the vasculitides, I agree that the differentiating diagnostic features are important.

I have read this submission. I believe that I have an appropriate level of expertise to confirm that it is of an acceptable scientific standard, however I have significant reservations, as outlined above.

# Response

**Published:** 2018-03-16

**Authors:** Saeid Jokar

**Affiliations:** yasouj university of medical sciences, Iran

Hi Patricia

 I corrected the article in accordance with your comments.

 Thank you for advising us on improving the content of the article.

 Best Regards

# Peer review: Referee response for version 1

**DOI:** 10.5256/f1000research.14428.r29766

**Published:** 2018-01-25

**Authors:** Marco de Vincentiis

**Affiliations:** 1 Department of Oral and Maxillofacial Sciences, Sapienza University of Rome, Rome, Italy

The authors present a case of a 65 year old woman that was admitted to the authors' hospital due to abdominal pain and skin lesion on the right upper and right lower extremities, with negative rheumatologic tests. After careful diagnostic workout, the final diagnosis was vasculitis (Polyarteritis nodosa or complicated Henoch-Schonlein purpura). The paper is interesting, also due to the rarity of the disease. However, there are some points that need to be improved before final approval:

1. The authors report Bell's Palsy in patient's medical history; it would be interesting to know when this occurred. Since this was treated with corticosteroid therapy, it is important to know if it was present when the patient already had PAN lesions - and in this case if they also improved during therapy - or if it occurred prior to PAN-related lesions arose.
2. Timing details are missing: in the case presentation - follow up and outcomes - the authors report that the patient "is being treated with 50mg prednisolone daily and then we will taper this amount". It is important to define for how long the patient is being treated, how much time past from initial diagnosis and therapy, and how the authors intend to reduce treatment. 
3. Sensorineural Hearing Loss is often reported in PAN and, in some cases, may occur as the presenting symptom. Hearing Loss is typically bilateral and symmetrical, with sudden or rapidly progressive onset. It would be interesting to know if hearing impairment was reported by the patient, or investigated. 
4. In the discussion we recommend to further discuss the findings that led to PAN diagnosis in this case.
5. The paper has several English language typos and grammar mistakes that should be corrected by a native English speaker (i.e. "which were was mostly", etc).

I have read this submission. I believe that I have an appropriate level of expertise to confirm that it is of an acceptable scientific standard, however I have significant reservations, as outlined above.

## References

[1] De VirgilioAGrecoAMagliuloGGalloARuoppoloGConteMMartellucciSde VincentiisM: Polyarteritis nodosa: A contemporary overview. *Autoimmun Rev*.2016;15(6) : 10.1016/j.autrev.2016.02.015 564-70 10.1016/j.autrev.2016.02.015 26884100

# Response

**Published:** 2018-01-29

**Authors:** Saeid Jokar

**Affiliations:** yasouj university of medical sciences, Iran

Hi Marco,

Thank you for attention in review of our article. I will correct the article by your statements.

1. One week pre-admission the patient was under corticosteroid treatment  because bell's palsy.
2. After 2 weeks, if the no recurrent of patient symptoms we will taper off corticosteroids amount 10 %. We will reduce the dose of corticosteroids until reaches to control of patient symptoms, then by the patient condition will decide about it.
3. Thank you - I add your statement to the manuscript

Other comments are considered.
